# Biomechanical performance of a novel zero-profile interbody cage: A cadaveric study

**DOI:** 10.1371/journal.pone.0317375

**Published:** 2025-04-29

**Authors:** Jia Zhu, Yangyang Cui, Hangkai Shen, Zhenhua Liao, Hongsheng Gu, Weiqiang Liu

**Affiliations:** 1 Tsinghua Shenzhen International Graduate School, Tsinghua University, Shenzhen, China; 2 Department of Mechanical Engineering, Tsinghua University, Beijing, China; 3 Biomechanics and Biotechnology Lab, Research Institute of Tsinghua University in Shenzhen, Shenzhen, China; 4 China United Engineering Corporation, Hangzhou, China; 5 Department of Spine Surgery, The Second People’s Hospital of Shenzhen, Shenzhen, China; PGIMER: Post Graduate Institute of Medical Education and Research, INDIA

## Abstract

Zero-profile cage (ZPC) products have been widely used in anterior cervical decompression and fusion (ACDF) surgery. To develop a ZPC that meets the biomechanical requirements of the Chinese population, we designed a novel zero-profile cage (NZ) by analyzing the critical anatomical parameters of the cervical spine in healthy Chinese people. This study aims to investigate and assess whether the biomechanical properties of the newly designed NZ could satisfy the criteria for clinical application. The biomechanical properties of the NZ were evaluated by being implanted into cervical cadaveric specimens, measuring and analyzing the range of motion (ROM) of surgical segments. The experimental group in this study consisted of the NZ. As the control group, the gold standard product combination of ACDF surgery, anterior fixation plate combined with cage (P + C), and the FDA-approved ZPC product (Zero-P) were utilized. The experiment utilized six cadaveric specimens of human cervical vertebrae subjected to identical testing conditions. Following the completion of the test under intact conditions, fusion products were implanted into each specimen in segment C4-C5 in the following order: Zero-P, NZ, P + C. Biomechanical results revealed that the ROM of the surgical segment had decreased significantly under six basic working conditions following NZ implantation. Statistically significant differences were observed in the left bending (LB), right bending (RB), and left rotation (LR) conditions when compared to the intact conditions. The remaining working conditions did not exhibit a significant difference. However, the observed decreasing trend was consistent with previously documented research. In terms of the ROM of surgical segments, there was no statistically significant difference between the NZ group, the Zero-P group, and the P + C group. The biomechanical properties of the newly designed NZ in this study were superior, comparable to the fusion effect observed in conventional products of the Zero-P group and the P + C group. Furthermore, the biomechanical properties exhibited further improvement when subjected to LB and RB conditions. In the future, the newly designed NZ has great potential as a competitive choice for clinical applications.

## Introduction

Cervical spondylosis arises from the degeneration and subsequent alterations of the cervical intervertebral disc. It is a clinical symptom and sign manifested when the nerve roots, spinal cord, blood vessels, esophagus and other related adjacent tissues are stimulated or compressed by the degenerated intervertebral disc [1]. The treatment of cervical spondylosis includes both non-surgical and surgical approaches, a significant proportion of patients may require surgical intervention [2].

Anterior cervical decompression and fusion (ACDF) is the gold standard surgical procedure for treating intervertebral disc herniation. The traditional surgical product of anterior fixation plate combined with cage (P + C) is to remove the degenerated cervical intervertebral disc and implant a fusion device to relieve the compression of the protruding intervertebral disc on the spinal cord and nerve roots [3]. The anterior plate could fix the surgical segment, prevent the withdrawal, misplacement and subsidence of the bone graft or cage [4–7]. However, the plate could also cause adjacent segment ossification, resulting in symptoms like the sensation of a foreign object in the throat, difficulty swallowing, tracheal injury, and upper airway obstruction [8–11]. Zero-profile cage (ZPC) products, on the other hand, could minimize the impact on adjacent soft tissues, reducing the occurrence of swallowing difficulties and other complications associated with plate protrusion. Additionally, ZPC products exhibited sustained clinical efficacy and a high fusion rate [12]. In ACDF surgery, they function as a viable substitute for the conventional P + C product.

The ZPC products utilized in Chinese market predominantly are composed of products that have been specifically designed and manufactured for the European and United States markets, such as ROI-C (ZimVie Inc., Broomfield, CO, USA), COALITION (Globus Medical Inc., Audubon, PA, USA), Zero-P VA (DePuy Synthes, Inc., Warsaw, IL, USA), STALIF-C (Centinel Spine, Inc., West Chester, PA, USA), etc. Due to notable variations in the typical anatomical parameters of the cervical spine between European and American populations and Asian populations, the biomechanical characteristics of the product may not attain the original design objectives when utilized in Asian and African patients, as compared to its application in European and American populations. Therefore, we have finalized the development of a novel zero-profile cage (NZ) designed for the Chinese population, taking into consideration the anatomical parameters of the cervical spine in healthy Chinese people.

The objective of this study is to assess the in vitro biomechanical properties of the newly developed cage NZ using the cadaveric specimen experiment. The biomechanical performance of the NZ will be compared with that of the ZPC product approved by Federal Drug Administration (FDA) and the generally recognized P + C product to ensure the validity of the biomechanical performance of the NZ when it is applied in the clinic. We hope to advance the promotion of ZPC products intended for the Chinese population in the field of clinical practice.

## Materials and methods

### Ethics statement

This study utilized exclusively cadaveric specimens devoid of any personally identifiable information. No cadaveric tissues or cells were used for clinical research. The Ethical Review Committee of Tsinghua University concluded that this study did not require ethical approval based on the published Approaches to Ethical Review of Research in the Life Sciences and Medicine Involving Human Beings in China. Informed consent was obtained and signed by the next of kin for the use of cadaveric specimens.

### Specimen preparation

We prepared six fresh-frozen human cadaveric cervical spine specimens (2 females and 4 males, mean age 50 (range, 33–70) years) for this study. They were exclusively preserved within a dedicated low-temperature freezer maintained at -20°C. A period of twelve hours preceding the preprocessing phase was allocated to retrieve the specimens and initiate the thawing process, which was accomplished by immersion in a 70% ethanol solution. Subsequently, following the complete thawing of the specimens, we underwent X-ray scanning, encompassing both anterior-posterior and lateral projections. This meticulous examination aimed to ascertain the integrity of the internal anatomical structures, including the identification of latent pathological conditions that elude naked-eye observation. Such conditions encompassed vertebral fractures, vertebral fissures, intervertebral disc herniation, intervertebral space narrowing, osteophyte formation, misalignment, and the loss of physiological curvature. Notably, any specimen displaying any of the aforementioned pathologies was categorically designated as non-compliant and thus excluded from further participation in the testing procedures.

To commence, we initiated the procedure by demarcating the targeted surgical segments, spanning from C_2_ to T_1_, on the cadaveric cervical spine specimens, employ-ing marking pins. Subsequently, a scrupulous dissection ensued, entailing the excision of superfluous spinal segments, notably C_1_ and certain portions of the thoracic vertebrae. This procedure was meticulously executed while preserving all essential anatomical components, including ligaments, joint capsules, intervertebral discs, and osseous structures imperative for the experimental setup [5,10,13].

Following this preparatory phase, we proceeded to affix three self-tapping screws into the vertebral bodies situated at the upper extremity of C_2_ and the lower terminus of T_1_. These screws were strategically positioned in a triangular arrangement. To secure and stabilize the specimens, a molten wood alloy was meticulously introduced into the mold, effectively encapsulating both the C_2_ and T_1_ vertebral bodies within the confines of the metal mold. This meticulous process served to ensure a robust and secure attachment of the cadaveric specimens to the upper cover and base of the testing apparatus in the final phase, we introduced perforations into the spinous processes of the casted vertebral bodies within the specimens. Subsequently, threaded rods were inserted into these perforations to facilitate the installation of infrared light-emitting position markers. Additionally, as a precautionary measure to avert specimen desiccation during the duration of the testing process, a saline solution was methodically sprayed onto the surface of the specimens at intervals of every 5 minutes [13,14].

### Biomechanical testing

**Fig 1:**
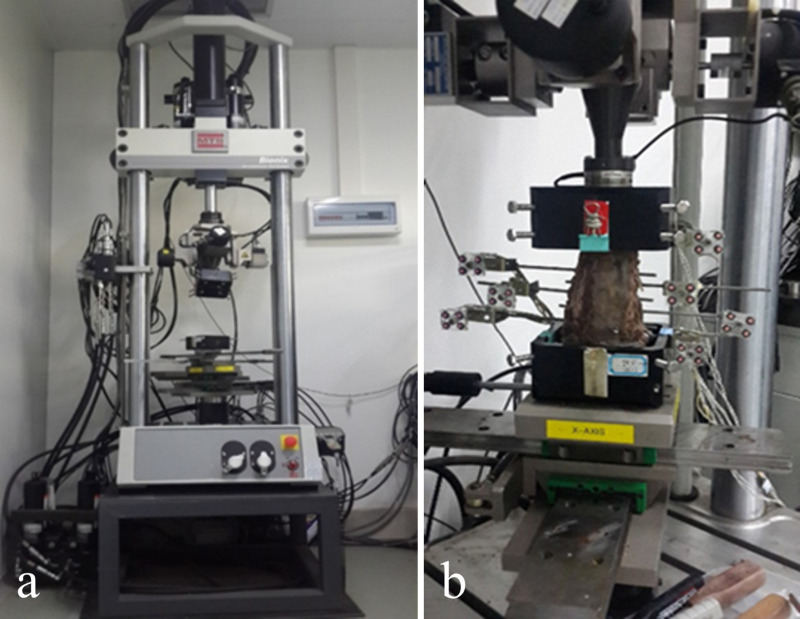
Initial preparation state diagram of the specimen experiment. a Spinal motion simulator MTS. b Intact specimen fixed on the spinal motion simulator with installed infrared light-emitting position markers.

The equipment employed in this experiment was the MTS Bionix 370.02A/T Spinal Motion Simulator (MTS Systems, Corp., Eden Prairie, MN, USA), which applied pure torque to the specimen via servo motors, thereby facilitating motion in six fundamental working conditions, including flexion (Flex), extension (Ext), left bending (LB), right bending (RB), left rotation (LR) and right rotation (RR) (Fig 1).

To mitigate the viscoelastic effects of the spine, each test specimen underwent three loading-unloading cycles in each physiological plane, generating displacement curves under flexion-extension, lateral bending to the left and right, and left and right axial rotation loads. Data from the third cycle were considered as valid data for collection, which were subsequently utilized for the final data analysis. The real-time motion coordinates of each individual vertebral body were meticulously recorded by employing the Optotrak Certus motion analysis system (Northern Digital Inc., Waterloo, Ontario, Canada) with infrared light-emitting position marker rigidly coupled to each vertebral level.

The hybrid testing methodology, introduced by Panjabi [15] and grounded in the principles of flexibility and stiffness principles, presents a pioneering testing paradigm widely embraced, acknowledged, and implemented in in vitro biomechanical assessments, offering conspicuous advantages. The experimental loading protocol employed in this investigation adhered to the hybrid testing approach and was delineated as follows:

a. Positioned the cervical spine specimen, equipped with the ability for motion redistribution, in a neutral stance, with the upper and lower ends securely fixed.
b. Administered a judicious, non-restrictive pure torque in accordance with the standard flexibility methodology. Employed a load control scheme to systematically apply a maximum torque of 1.5 Nm at a rate of 0.1°/s [16,17]. Measured and recorded the comprehensive range of motion (ROM) of the specimen under its intact conditions.
c. Subsequent experimental phases employed the stiffness methodology to apply forces to the cervical spine specimen. In other words, loaded the specimen by employing a displacement control scheme until its actual ROM aligned with the overall ROM recorded in b. Throughout this procedural sequence, documented and archived the positional dynamics of each vertebral body within the specimen [18,19].

All cadaveric specimens underwent a secondary visual inspection and X-ray ex-amination following the completion of surgery to ensure accurate implant positioning and to eliminate the possibility of specimen damage resulting from the implantation procedure. Furthermore, after completing the biomechanical testing, a thorough ex-amination of both specimens and implants revealed no evidence of specimen damage or indications of loosening or fracture of implant screws.

### Study design

**Fig 2:**
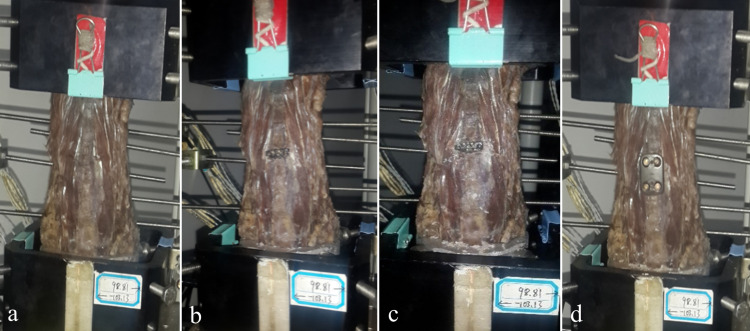
Testing states of cadaveric specimens under different surgical conditions. a Intact. b Zero-P. c NZ. d P + C.

To control for variability between specimens, each cadaveric specimen underwent testing at the C_4_-C_5_ segment in the following sequence (Fig 2): (1) Intact (n = 6); (2) Discectomy, decompression, and implantation of the FDA-approved zero-profile cage (Zero-P) (n = 6); (3) Utilization of the NZ specifically designed for the Chinese population (n = 6); (4) Product P + C licensed by the FDA (n = 6). Furthermore, all surgical procedures were executed by certified spinal surgeons.

**Fig 3:**
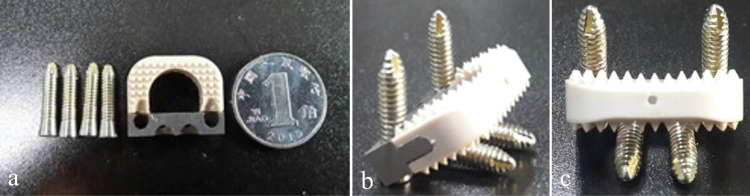
Design and machining schematic of the novel ZPC for the Chinese population. a Top view. b Isometric view. c Rear view.

The testing apparatus for the experimental group in this study was the NZ specially designed for the Chinese population. Its shape was illustrated in Fig 3, and the design dimensions were provided in Table 1. The NZ consisted of three main components: the polyetheretherketone (PEEK) fusion body, the titanium alloy zero-profile fixation plate, and titanium alloy securing screws. The fusion body and fixation plate were connected through a mechanical structure, while the securing screws and fixation plate were threadedly connected to maintain appropriate compression force. Four securing screws were drilled into the vertebral bodies at different angles, achieving short-term fixation of the implant to the vertebral bone. Regarding the products utilized in the control group, the Zero-P^TM^ (DePuy Synthes, Inc., Warsaw, IL, USA) was the zero-profile anterior cervical interbody fusion device employed in the ZPC group, featuring a 4-screw design. For the P + C group, the anterior cervical plating system used was the DOC Cervical Plate (DePuy Acromed, Inc., Mountain View, CA, USA). The cage utilized was a PEEK interbody fusion device, namely PLATEAU®-C (Life Spine, Inc., Huntley, IL, USA). Data were normalized, and the ROM for each segment was presented as a percentage of the total ROM for the cervical spine segment.

**Table 1:** Parameters of the newly designed ZPC for the Chinese population used in test.

Type	Footprints (Transverse × Sagittal)/ (mm × mm)	Height H/mm	Curved radius R/mm	Screw Length L/mm
S	14.5 × 11.5	3.5, 4, 5, 6	9.1	12, 14
M	15.5 × 12.5	4, 5, 6	10.7	12, 14, 16

### Statistical analysis

The software Matlab (version 2021a; MathWorks, Natick, MA, USA) was utilized to develop the data processing program. The 3dx.format file obtained from the Optotrak Certus motion analysis system was processed so as to obtain the coordinates of the maximum absolute position of each vertebral segment and as well as the percentage of the ROM of each segment over the ROM of the whole segment. In essence, the data were normalized, and the specific calculation principle could be expressed as follows: ω_ROM_ = 100 × S_ROM_/T_ROM_, where S_ROM_ represented the ROM of a specific segment, T_ROM_ represented the ROM of the entire cervical spine segments from C_2_ to T_1_, and ω_ROM_ represented the percentage of the ROM of a single segment in the overall ROM of the cervical spine [15,19].

Statistical analysis was performed on the percentage values of the ROM for each segment of each specimen under various working conditions, which were outputted from Matlab. Excel (version 2023; Microsoft Corp, Redmond, WA, USA) software was utilized to calculate the mean value of the percentage and the standard deviation. The aforementioned data were subsequently represented in the form of histograms for visual statistical analysis using Origin (version 2023; OriginLab, Hampden, MA, USA) data processing software. One-way analysis of variance (One-way ANOVA) was conducted between the experimental group and the control group using IBM SPSS Statistics software (version 26.0; IBM Corp, Armonk, NY, USA) [20]. A statistically significant difference was deemed to exist between the two data groups when the ANOVA significance level was set at p < 0.05; conversely, no statistically significant difference was considered between the two groups of data [20,21].

## Results

### ROM distribution of all segments in intact specimen

**Fig 4:**
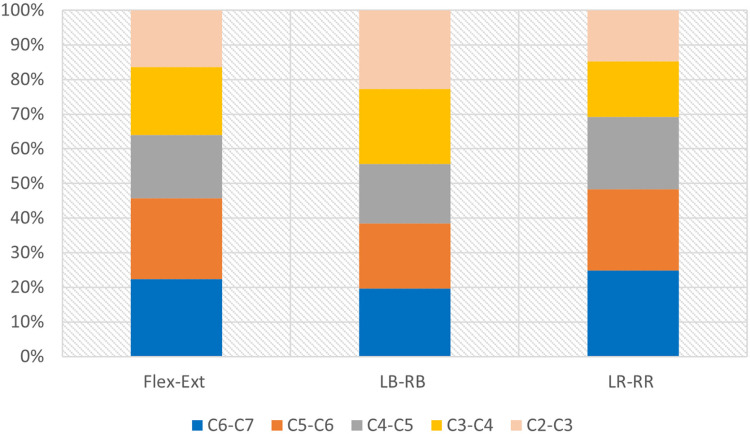
Stacked bar of motion distribution of intact specimen under different working conditions.

This biomechanical test was performed using a hybrid test method, applying a non-restrictive pure moment loading method. The distribution of ROM in each segment of the intact specimen reflects whether the loading method is correct or not, which in turn determines whether the test results are reliable or not. The ROM distribution data had been normalized and expressed as percentage values (as shown in Fig 4 and Table 2).

**Table 2:** Summary of ROM percentage for each segment in intact specimen.

Group	C2-C3	C3-C4	C4-C5	C5-C6	C6-C7
Flex-Ext (%)	16.34	19.67	18.21	23.43	22.36
LB-RB (%)	22.77	21.58	17.23	18.81	19.61
LR-RR (%)	14.82	16.33	21.04	23.76	25.05

### ROM comparison of intact specimen with all test groups

**Fig 5:**
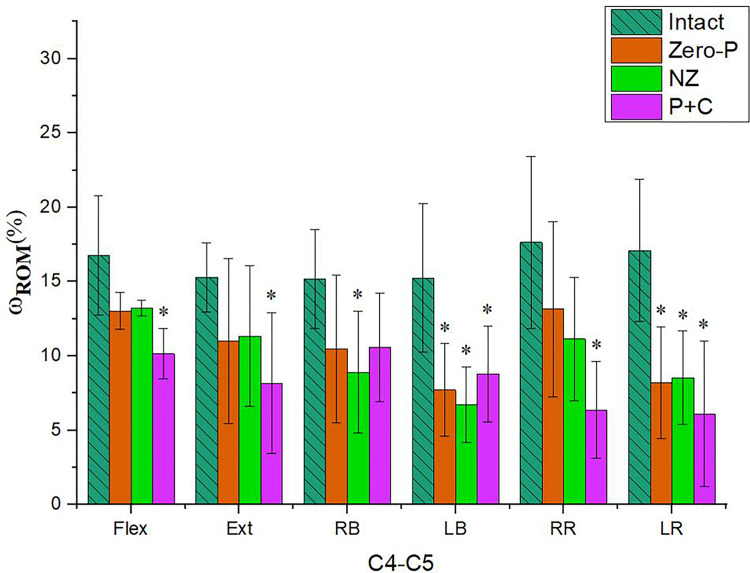
Histogram of motion distribution of specimens after different surgical products under six basic working conditions. (* represents statistically significant differences p < 0.05, Ι represents the value of standard deviation of the data).

Surgical procedures were conducted at the C_4_-C_5_ segment, and the pre-operative and post-operative ROMs of cervical spine specimens under six fundamental working conditions were presented in Fig 5 and Table 3. The implantation procedures experienced by the specimens were Intact, Zero-P, NZ, and P + C. The results were described in detail below.

**Table 3:** Summary of ROM normalization results for surgical segments after different surgeries for specimens under six basic working conditions.

C4-C5 ROM (Mean ± SD) %	Intact	Zero-P	NZ	P + C
	n = 6	n = 6	n = 6	n = 6
Flex	16.8 ± 4.02	13.0 ± 1.25	13.2 ± 0.52	10.2 ± 1.69
Ext	15.3 ± 2.33	11.0 ± 5.55	11.3 ± 4.73	8.2 ± 4.75
RB	15.2 ± 3.31	10.5 ± 4.99	8.9 ± 4.09	10.6 ± 3.63
LB	15.2 ± 4.99	7.7 ± 3.10	6.7 ± 2.53	8.8 ± 3.21
RR	17.7 ± 5.78	13.2 ± 5.89	11.1 ± 4.16	6.4 ± 3.25
LR	17.1 ± 4.78	8.2 ± 3.78	8.5 ± 3.14	6.1 ± 4.90

Under the flexion condition, the ROM of the surgical segment utilized the Zero-P device exhibited a decrease of 22.22% without statistical significance. For the NZ product, the ROM of the surgical segment was reduced by 21.01%, which was similar to the change in the Zero-P product and again not statistically significant. In contrast, the ROM with the P + C product decreased significantly by 39.43% (p = 0.018).

The ROM of the surgical segments was largely reduced after the implantation of all three products under the extension working condition. The P + C group exhibited the largest reduction of 46.51%, which was statistically significant (p = 0.024). The Zero-P and NZ groups showed similar reductions, which were smaller than the P + C group, with reductions of 27.91% and 25.79%, respectively. No statistically significant difference was observed in the reduction amplitudes of the two groups.

Under the RB working condition, the Zero-P group exhibited a reduction of 30.99%. Within the NZ product group, it was observed that the ROM experienced a reduction of up to 41.26%. This reduction was found to be statistically significant, with a level of significance value of p = 0.027. The surgical segmental ROM was found to be reduced by 30.31% in the P + C group. This reduction was similar to the Zero-P group, with their respective p-values of 0.085 and 0.092, indicating that neither of these two groups reached statistical significance.

In contrast, under the LB working condition. The postoperative ROM for the C_4_-C_5_ segment was significantly reduced by 42.41%, 55.80%, and 49.34%, respectively, for the Zero-P product group, the NZ product group, and the P + C group. The corresponding significance level values for these differences were p = 0.004, p = 0.002, and p = 0.011, respectively.

The Zero-P group showed a statistically significant difference (p = 0.003) with a 52.04% reduction in ROM under the LR working condition, while under RR working condition, the ROM was reduced by 25.42%, which was not statistically significant. The ROM of the NZ product group decreased by 50.14% under the LR working condition, indicating a statistically significant difference (p = 0.004). Conversely, the ROM decreased by 36.87% under the RR working condition, without showing a statistically significant difference. The ROM of the P + C product group decreased by 63.87% p and 64.27%, respectively, under RR and LR working conditions. Both exhibited statistically significant difference (p < 0.05), with p = 0.010 and p = 0.001, respectively.

### ROM comparison between all test groups

ROM comparisons between two test groups could reflect the level of variability in biomechanical properties of different products. ROM comparisons between any two groups were performed for the three products NZ, Zero-P and P + C used in this study. The significance levels of the three comparison groups at the six basic working conditions were summarized in Table 4 below. The smallest significance level is 0.10, which occurs in the comparison of the Zero-P and P + C products under the RR working condition. All significance levels were >0.05, and no statistically significant differences were demonstrated between any two groups under any of the basic working conditions.

**Table 4:** Summary of significance levels for comparison of surgical segment ROM between different test groups.

Significance level p	Flex	Ext	RB	LB	RR	LR
NZ—Zero-P	0.92	0.91	0.55	0.67	0.57	0.90
NZ—P + C	0.13	0.28	0.53	0.38	0.25	0.39
Zero-P—P + C	0.15	0.33	0.97	0.65	0.10	0.45

## Discussion

The primary objective of the present study was to assess whether the novel zero-profile fusion device NZ developed for the Chinese population possessed the necessary biomechanical properties to fulfill the requirements of clinical applications. Following the implantation of the NZ into the intervertebral space of the cervical spine cadaveric specimen, the ROM of the surgical segment was measured and analyzed to assess the postoperative matching effect, the fusion outcome, and the impact on the biomechanical stability of the surgical segment. By comparing the ROM of the NZ to the non-operative intact state of the cervical specimen, it was possible to ascertain whether the postoperative fusion results of the NZ were qualified. Additionally, to enhance the dependability of the experimental procedure and outcomes, the Zero-P product and the DOC anterior cervical fixation plate combined with the PLATEAU^®^-C cage product were chosen for this study to conduct single-segment ACDF surgeries under the same surgical conditions as the control group for this experiment. The P + C traditional surgical product group was clinically recognized as the gold standard for the treatment of cervical spondylosis, and the Zero-P product group was a clinically accepted ZPC product. Employing these two product groups as the control group for this study could effectively test whether the NZ reached the level of products widely recognized by the market and clinic, making the study more convincing.

A statistical investigation was carried out by Dvorak et al. [22] concerning the ROM of each segment during flexion and extension movements of the cervical spine. Penning [23] examined the ROM of each segment during LB, RB and flexion movements of the cervical spine. The ROM for each segment of the cervical spines were normalized as reference range. Based on the range, it was evident that the ROMs of C_2_-C_3_, C_3_-C_4_, and C_6_-C_7_ for this study fell within the reference range of ROM. The ROMs of C_4_-C_5_ and C_5_-C_6_ were 18.21% and 24.43%, which surpassed the range reference range 18.76%-23.08% and 24.10%-29.73%. However, the excess in both segments was less than 1%, and no statistically significant difference could be identified. Consequently, it could be proved that the measurement results under the flexion and extension working conditions were dependable when the test employed a mixed loading mode.

In previous researches, there were few studies testing the changes in the ROM of each segment under the bending working condition, except for the studies conducted by Panjabi et al [24] and Wei [25]. By conducting a comparative analysis, it was found that the current test followed the same trend as the two previously conducted tests, which both indicated a declining ROM for each surgical segment. This finding aligned with the established knowledge and conclusions of the previous studies. Furthermore, no statistically significant difference was observed among the three groups.

Moriya et al. [26] and Lai et al. [27] investigated the ROM of each segment during rotational movements of the cervical spine. Based on the results of ROM normalization, it was evident that the ROMs for segments C_2_-C_3_, C_4_-C_5_, and C_5_-C_6_ in the current study fell within the reference range of ROM. While the ROMs for C_3_-C_4_ and C_6_-C_7_ deviated from the reference range 19.86%-22.73% and 17.14%-21.92%, the discrepancy in values was not significant. Thus, the measurements of rotation remained dependable in the current loading mode. The ROM was found to be outside the expected range, potentially due to factors such as variations in the quality of the experimental specimen’s bone. The in vitro biomechanical test conducted by Panjabi et al. [24] also revealed that certain segments deviated significantly from the reference range. For instance, the ROM for segment C_4_-C_5_ under rotational conditions was 29.44%, surpassing the in vivo reference range. Nevertheless, the general trend of segment change was consistent with prior researches, and this study of Panjabi et al. [24] was still widely recognized as one of the most important basic studies in the field of biomechanics.

In the current study, it was evident from the outcomes of the motion distribution analysis that cadaveric specimens exhibited substantially reduced ROM in the surgical segment after the fusion procedure, regardless of the implanted product type. In the P + C product group, a significant reduction in the ROM of the surgical segment C_4_-C_5_ was noted after product implantation. Statistically significant differences (p < 0.05) were observed in all five working conditions (Flex, Ext, LB, LR and RR) when compared to the intact condition. This finding suggested that the P + C product underwent significant fusion effect under the five working conditions, which was consistent with the outcomes documented in prior research [28–32]. In an ideal scenario, fusion results for the surgical segments should show significant differences in all six basic working conditions. However, in the current study, despite a substantial reduction in ROM, the RB working condition failed to exhibit any significant differences. Matti et al. [33] obtained comparable outcomes in a multi-segmental investigation that deviated slightly from the ideal scenario. No significant differences were observed in any of the lateral bending working conditions, which were primarily ascribed to variations in the quality of the specimen and discrepancies in specimen handling or mounting during experimental preparation. Given the consistency of the observed trend with all prior investigations, it continued to possess value as a reference and for research endeavors.

Among the numerous investigations examining the ROM of surgical segments after ZPC implantation in cervical cadaveric specimens, Nayak et al. [34] successfully implanted three-screw ZPCs in two consecutive segments and revealed a statistically significant decrease in ROM for both surgical segments when compared to the intact condition. Additionally, in the flexion-extension condition, the P + C group exhibited a significantly smaller reduction in ROM than the ZPC group. Moreover, the P + C group showed a significantly greater reduction in ROM than the ZPC group under flexion-extension conditions, which was consistent with the results reported by Reis et al. [35]. Conversely, Arnold et al. [36] discovered that the blade-fixed ZPCs exhibited significant differences just under flexion-extension and lateral bending conditions, excluding rotational conditions. Both the screw-fixed and anchor-fixed ZPCs did not show significant differences under flexion-extension, lateral bending, and rotational working conditions. The Zero-P product group in this study exhibited statistically significant differences (p < 0.05) under the LB and LR working conditions after implantation of cervical cadaveric specimens. These results suggested that the Zero-P product demonstrated significant fusion efficacy under the two working conditions. While the observed results of ROM reduction might not completely align with the results of ideal surgical segments, which all demonstrated significant differences, they did follow the trend of ROM change observed in prior research. Following implantation of the newly designed NZ into the cervical cadaveric specimen utilized in this research, there were statistically significant differences (p < 0.05) in the ROM of the surgical segments under the three working conditions of LB, RB, and LR. This suggested that the NZ exhibited a significant biomechanical stability and fusion effect under the three working conditions. Additionally, it was observed that the experimental NZ group exhibited a comparable trend and pattern of change to the control Zero-P group. Moreover, under the RB working condition, the experimental NZ group demonstrated a more pronounced fusion effect and significant biomechanical stability compared to two control groups.

Comparing the ROMs of various implanted products to determine whether there were significant differences in biomechanical properties was an efficient method for demonstrating the consistency of biomechanical property levels across products. By comparing the ROM of various implant products, Scholz et al. [28] determined that the ZPC group, anterior fixed plate with cage, and dynamic anterior fixed plate with cage did not differ statistically significantly from one another in human cervical specimens. Based on this finding, the researchers concluded that the ZPC had biomechanical stability comparable to conventional anterior titanium plate fixation combined with cage implantation when applied to single segments. Comparing the ROM of surgical segment to that of intact condition, Majid et al. [29] found that anterior titanium fixation plate combined with cage and the ZPC significantly reduced ROM in surgical segment. And no significant difference in ROM was observed between these two types of products. In the same way, Stein et al. [30] evaluated a three-screw ZPC product and found no statistically significant difference in ROM reduction when compared to conventional anterior titanium fixation plate with cage. However, Reis et al. [35] obtained a divergent finding. The researcher discovered that the two-screw ZPC group experienced a comparatively greater reduction in ROM than the P + C group under statistically significant differences under the flexion, extension and rotation working conditions. Furthermore, this reduction was greater than that of the four-screw ZPC group, which demonstrated statistically significant differences in extension and rotation conditions as well. In contrast, under all working conditions, there was no statistically significant difference between the ROM values of the four-screw ZPC group and the P + C group. The results presented in Table 4 indicated that there was no significant difference between the Zero-P group, the NZ group, and the P + C group in terms of surgical segment ROM under the six fundamental working conditions. This suggested that the NZ developed in this study exhibited functional comparability to both the Zero-P product and the P + C product, particularly in regards to the postoperative biomechanical stability of the surgical segments.

Since it was originally a cervical spine cadaveric specimen model utilized for biomechanical performance research, it possessed some limitations. The physiological environment of the human cervical spine could not be exactly replicated in cadaveric specimens. Fresh frozen specimens merely offered a reasonably accurate representation of the environment, the muscle tissue from a cervical cadaver would have some degree of damage, and there were no skin moisturizing and thermal insulation conditions, which would provide some additional stability to the product in its natural physiological environment. Although the in vitro cervical spine cadaveric specimen experiment had its limitations, it still represented one of the most realistic experimental protocols during the product validation phase. It provided immediate information of critical parameters including ROM, motion distribution, and intervertebral space height of the cervical spine after surgery. Clinicians and experts in the field of research have consistently acknowledged and accepted this. Therefore, we argued that the experimental results of this study could provide valuable insights for the selection of cervical single-segment fusion protocols.

## Conclusions

Through a comprehensive analysis of the test data for the present study, we had found the following. The biomechanical properties of the newly designed NZ in this study were qualified, which could result in a substantial reduction in surgical segmental ROM under all six fundamental working conditions. Comparable biomechanical properties were observed between the NZ and clinically recognized conventional products, and there was no statistically significant difference in postoperative ROM among the three product groups—the Zero-P group, the NZ group, and the P + C group. Furthermore, the biomechanical performance of the NZ was superior to that of the Zero-P and P + C products under LB and RB working conditions, while it was comparable under all other conditions. In summary, the biomechanical properties of the NZ designated for the Chinese population were comparable to those of clinically recognized products, and even showed some superiority under some working condition.

While our current study has validated that the biomechanical properties of the new product are comparable to FDA-approved alternatives in single-segment applications using cadaveric specimens, we recommend that further research should be pursued to ensure that the comprehensive performance of the product also satisfies clinical application requirements. Future work should include a comparative analysis of multi-segmental surgical procedures in vitro specimens, aiming to highlight the comprehensive biomechanical properties of the product and enrich its potential clinical application scenarios in the future. Additionally, animal experimentation for in-vivo assessment serves as a valuable method for biomechanical evaluation. They allow for the assessment of the product’s impact on surgical segments and adjacent segments in an environment that closely mimics natural applications, thereby accumulating highly credible experience for future clinical use.

## Supporting information

S1 Dataset Raw data. (XLSX)
